# Draft Genome Sequence of *Stenotrophomonas maltophilia* Strain 5BA-I-2, a Soil Isolate and a Member of a Phylogenetically Basal Lineage

**DOI:** 10.1128/genomeA.00134-14

**Published:** 2014-03-06

**Authors:** Jaroslav Nunvar, Dana Elhottova, Alica Chronakova, Bohdan Schneider, Irena Licha

**Affiliations:** a Department of Genetics and Microbiology, Faculty of Science, Charles University, Prague, Czech Republic; b Institute of Biotechnology of the ASCR, Prague, Czech Republic; c Institute of Soil Biology, Biology Centre ASCR, Ceske Budejovice, Czech Republic

## Abstract

*Stenotrophomonas maltophilia* is an omnipresent environmental bacterium emerging as an opportunistic human pathogen and exhibiting multidrug resistance. Here, we report the draft genome sequence of *S. maltophilia* strain 5BA-I-2, a soil isolate and a member of a phylogenetically basal lineage.

## GENOME ANNOUNCEMENT

*Stenotrophomonas maltophilia* is a gammaproteobacterial species that is closely related to xanthomonads (1). Strains of this species are frequently isolated from water and soil habitats, including the rhizosphere (2). This bacterium shows significant potential for biocontrol of a variety of plant diseases, as well as for the remediation of chemically diverse xenobiotics (3). Furthermore, *S. maltophilia* can readily infect predisposed humans, mostly those who are immunocompromised and patients suffering from cystic fibrosis (4–7). These infections are difficult to treat due to intrinsic resistance to multiple antibiotics, a common attribute of the environmental and clinical strains (8). Despite a common pattern of multiresistance, the overall genetic variability of the species was found to be considerably high (9, 10).

*S. maltophilia* 5BA-I-2, previously referred to as strain env2 (11), was isolated from grassland soil in southern Bohemia (Czech Republic) in July 2007, and the source strain was deposited in the Institute of Soil Biology, Biology Centre (BC), Academy of Sciences of the Czech Republic (ASCR), in Ceske Budejovice.

The genome sequence was determined on the 454 GS Junior system (Roche) (12). A total of 185,523 reads comprising 83,879,639 bases were assembled using the Roche GS *de novo* assembler (version 2.7) and Geneious R6 assembling software (version 6.1.6). The combination of these independent assembly methods yielded four contigs totaling 4,568,054 bp, which were entirely collinear with the complete genome of the closely related strain *S. maltophilia* R551-3 (13). The contigs were submitted to the NCBI Prokaryotic Genome Annotation Pipeline version 2.0. The annotation predicted 4,162 genes, consisting of 4,056 coding sequences, 27 pseudogenes, 12 rRNAs, 66 tRNAs, and 1 noncoding RNA (ncRNA). A multitude of putative antibiotic resistance and efflux system genes are present, as is observed in other strains of the species. Repetitive extragenic palindromic (REP) elements of the SM4, SM8, and SM10 classes (14) are the most abundant types of repeats in the *S. maltophilia* 5BA-I-2 genome.

The nucleotide sequences of seven multilocus sequence typing loci (15) placed *S. maltophilia* 5BA-I-2 in genogroup 9, which is synonymous with the 16S rRNA E2 group (16, 17). This distinct phylogenetic lineage is clearly basal with respect to other genomic groups within the *S. maltophilia* species, and it contains only isolates of environmental origin (17, 18). As the first genome sequence for a member of genogroup 9, the genome sequence of *S. maltophilia* 5BA-I-2 will prove valuable in comparative and evolutionary genomics studies.

### Nucleotide sequence accession numbers.

This whole-genome shotgun project has been deposited at DDBJ/EMBL/GenBank under the accession no. AZAE00000000. The version described in this paper is the first version, AZAE01000000.
